# Neural Stimulation Has a Long-Term Effect on Foreign Vocabulary Acquisition

**DOI:** 10.1155/2015/671705

**Published:** 2015-05-14

**Authors:** Achille Pasqualotto, Begüm Kobanbay, Michael J. Proulx

**Affiliations:** ^1^Faculty of Arts and Social Sciences, Sabanci University, Tuzla, 34956 Istanbul, Turkey; ^2^University of Bath, Bath BA2 7AY, UK; ^3^Queen Mary University of London, London E1 4NS, UK

## Abstract

Acquisition of a foreign language is a challenging task that is becoming increasingly more important in the world nowadays. There is evidence suggesting that the frontal and temporal cortices are involved in language processing and comprehension, but it is still unknown whether foreign language acquisition recruits additional cortical areas in a causal manner. For the first time, we used transcranial random noise stimulation on the frontal and parietal brain areas, in order to compare its effect on the acquisition of unknown foreign words and a sham, or placebo, condition was also included. This type of noninvasive neural stimulation enhances cortical activity by boosting the spontaneous activity of neurons. Foreign vocabulary acquisition was tested both immediately and seven days after the stimulation. We found that stimulation on the posterior parietal, but not the dorsolateral prefrontal cortex or sham stimulation, significantly improved the memory performance in the long term. These results suggest that the posterior parietal cortex is *directly* involved in acquisition of *foreign* vocabulary, thus extending the “linguistic network” to this area.

## 1. Introduction

The acquisition of knowledge is a topic that has long fascinated classical philosophers [1] and early experimental psychologists [2]. Now, as people tend to travel more for work and/or leisure [3, 4], acquisition of a particular type of knowledge has become extremely important: the acquisition of foreign languages. For example, knowing a second language often increases the possibility of finding a better job or it can improve one's career [5, 6]. Language learning, even in older adults, has also been found to have additional health benefits by delaying dementia [7]. However, due to the large number of items, acquisition of the vocabulary of a foreign language is extremely costly in terms of time, energy, and often money. Several efforts have been made to improve general knowledge acquisition, for example, by creating new learning methods [8], new learning tools [9], and new learning strategies [10]. However, very few studies have attempted to investigate the effectiveness of noninvasive electric brain stimulation for linguistic acquisition [11], and no study has attempted to use transcranial random noise stimulation (tRNS). In fact, tRNS has only become available in recent times [12], and so far it has been sparsely employed, for example, in conjunction with basic perceptual processing [13] or numerical competence [14, 15]. tRNS increases neural activity by acting on sodium channels [12, 16] and possesses advantages compared to other types of brain stimulation, such as transcranial direct current stimulation (tDCS). In fact, it has been demonstrated that tRNS is less likely to produce side effects such as “itchy,” “pulling,” or “burning” sensations or even to be detected [17]. Moreover, brain stimulation is an important method to determine whether cortical regions are causally related in a particular function, rather than having just correlated activity as assessed by brain imaging techniques [18].

Aside from the “classical” language areas of Broca and Wernicke [19, 20], linguistic processing and production are also associated with other areas of the temporal lobe (e.g., lateral and anterior temporal cortex), auditory cortex, and basal ganglia [21–26]. Yet, it is still debated whether this “linguistic network” is actually larger and encompasses neighbouring areas, such as the posterior parietal cortex [24] and the dorsolateral prefrontal cortex (DLPFC [27]). The posterior parietal cortex possesses extensive connections with* posterior* language areas (e.g., Wernicke's area [28]) and is likely to be involved in linguistic processing [24]. On the other hand, DLPFC is well known for its role in working memory function [29, 30]; thus it is likely to play a role in the memory consolidation of foreign words. Additionally, DLPFC is extensively connected with* anterior* language areas (e.g., Broca's area [28]).

In this study, we* directly* investigated the causal role of these brain areas in foreign vocabulary acquisition by using* bilateral* transcranial random noise stimulation. To control for experiment-induced artifacts, we included a sham (placebo) group. We examined both the* immediate* and* long-term* effects of tRNS; however, we were particularly interested in investigating the* long-term* effect of brain stimulation on foreign language acquisition, since previous studies reported a long-term effect of brain stimulation on numeric competence [16, 31]. During the stimulation, participants performed a foreign vocabulary learning task, where they learnt Swahili words to which they had no prior exposure [32]. The long-term effect of brain stimulation on foreign vocabulary acquisition was assessed in a testing session held seven days after the main session, and here participants did not receive any stimulation. Based on the findings of previous studies using different techniques (e.g., [24, 27]), we expected to find that, compared to sham stimulation, tRNS on the frontal or the parietal areas would reveal which areas produce long-term benefit (e.g., [16]) in foreign vocabulary acquisition. 

## 2. Material and Methods

### 2.1. Participants

We tested 54 participants (27 males and 27 females) recruited among the students of Queen Mary University of London. Every participant completed an exclusion questionnaire ensuring that none of them was affected by or had history of neurologic, psychiatric, or systemic pathologies incompatible with brain stimulation (e.g., epilepsy); that nobody had history of substance abuse; that nobody was under any drug treatment acting on the central nervous system; and that nobody was affected by motor impairments. One participant who reported to be affected by migraines was excluded from testing. No participant had damaged skin over the scalp, and no participant had been exposed to Swahili before. All participants had normal or corrected-to-normal vision and signed the consent form approved by Queen Mary University of London Research Ethics Committee and the University of Bath Psychology Research Ethics Committee. Each experimental group was composed of 18 participants (nine males and nine females). The group that received frontal stimulation had an average age of 21.17 (SD = 3.11) years, the parietal group had an average age of 21.28 (SD = 2.56) years, and the sham group had an average age of 22 (SD = 4.38) years.

### 2.2. Apparatus and Procedure

**Figure 1:**
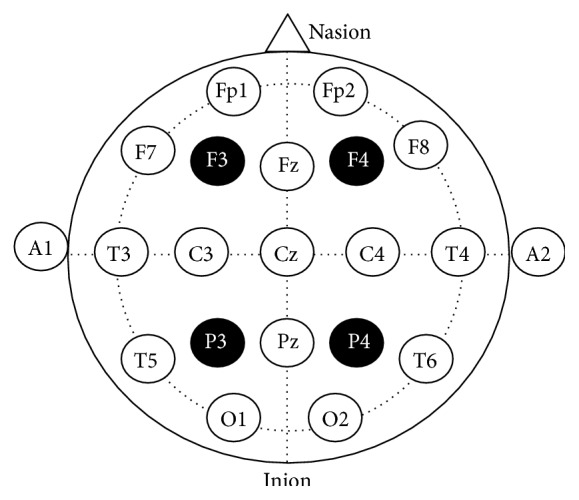
Depiction of the international 10–20 system; filled circles indicate the electrode positions used in this study.

The experiment took place over two different sessions with a 7-day gap in between. In the first session, participants completed the exclusion questionnaire, signed the consent form, had electrodes placed on their heads, and performed the foreign vocabulary learning task. After seven days, participants were tested on the material they had previously learnt; here* no* brain stimulation was applied. tRNS was delivered by a DC Stimulator Plus device (neuroConn GmbH, Germany) through two 5 × 5 cm electrodes inserted into saline-soaked synthetic sponges. Stimulation was bilateral and consisted of high frequency noise (100–600 Hz) with an intensity of 1 mA. Participants were randomly assigned to one of three groups: the frontal group received bilateral tRNS on the dorsolateral prefrontal cortex (areas F3 and F4 of the international 10–20 system (the head of each participant was measured along the two main axes (left-right and front-back), and the coordinates of the DLPFC and posterior parietal cortex were calculated) [33]) and the parietal group received bilateral tRNS on the posterior parietal cortex (areas P3 and P4); see Figure 1. For the frontal and parietal groups, stimulation was applied for 25 minutes with increasing and decreasing “ramps” of 10 seconds at the beginning and end. By following an established control protocol [12, 15, 30], participants in the sham group had electrodes placed on their heads for 25 minutes (bilaterally on the DLFPC or bilaterally on the posterior parietal cortex) but the actual stimulation lasted 20 seconds only (i.e., ramps only).

In the first experimental session, participants learnt 40 pairs of Swahili-English words that were presented on a computer screen using e-Prime (Psychology Software Tools Inc., USA). The 40 pairs of words used in this study were adopted from the study by Karpicke and Roediger III [32]; however, here we replaced English words with multiple spellings (e.g., “honour” and “honor”) with uniquely spelled words (see Table 1). During the session, each participant underwent four study-test blocks. In the study part of the block, participants were sequentially presented with Swahili-English pairs (e.g., “roho = soul”); each pair was visible for 5 seconds, and after each pair appeared a fixation-cross for 1 second. In the test part of the block, Swahili words were presented alone and participants were instructed to type the corresponding English translation (e.g., “roho = —”). Participants had a fixed period of 8 seconds to type, and feedback was not provided. Crucially, the drop-out method was employed in the task, which meant that the correctly remembered words were not studied or tested in the subsequent blocks. That is, in the first block, 40 pairs of words were studied and tested, but in the second block only the words that were not correctly recalled in the first block were studied and tested; in the third block only the words that were not correctly recalled in the first and second blocks were studied and tested; and in the fourth block only the words that were not correctly recalled in the first, second, and third blocks were studied and tested. This learning method was found to be time-efficient but to result in poor memory performances [32]; it was chosen to better highlight the effect of brain stimulation (i.e., more “room” to improve performance) and to avoid ceiling effects. After each of the four study-test blocks, participants performed an unrelated distractor task (number discrimination) for 1 minute.

Seven days after the first experimental session, the second experimental session took place, in which participants were presented with the 40 Swahili words and were asked to type the English equivalent (no tRNS). The first experimental session took about 35 minutes to complete, while the second session lasted about 15 minutes.

## 3. Results

**Figure 2:**
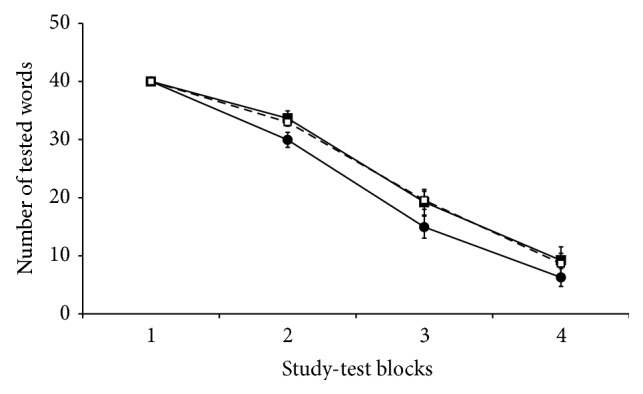
Average number of presented word pairs in the four experimental blocks (first/main session). All participants started with 40 pairs and, as participants correctly remembered them, the number of presented pairs decreased. Filled circles indicate parietal stimulation, filled squares indicate frontal stimulation, and empty squares indicate sham stimulation. Error bars represent the ±SEM.

We counted the number of words presented in each study-test block in the first experimental session for each participant; as the correctly recalled words were not presented in the following blocks, the number of presented words was used to measure memory performance (the fewer words presented indicated the greater memory performance). On this dataset we ran a two-way analysis of variance (ANOVA) with stimulation (frontal, parietal, or sham) and block (1st, 2nd, 3rd, or 4th) as independent variables. Although tRNS seems to have produced slightly better performance in the parietal group (see Figure 2), the effect of stimulation was not significant (*F*(2, 51) = 2.03, *p* = 0.14). The effect of block was highly significant (*F*(3, 50) = 467.7, *p* = 0.00), which indicates that as sequential study-test blocks proceeded, participants studied and were tested on fewer words (i.e., overall memory performance improved). The interaction stimulation by block was not significant (*F*(6, 47) = 1.33, *p* = 0.25), indicating that, regardless of the type of brain stimulation, participants improved their performance over the four study-test blocks (see Figure 2). Thus, learning performance in the first experimental session was similar across the three groups; thus any differences after the long-term delay would not arise from group differences in the first session.

**Figure 3:**
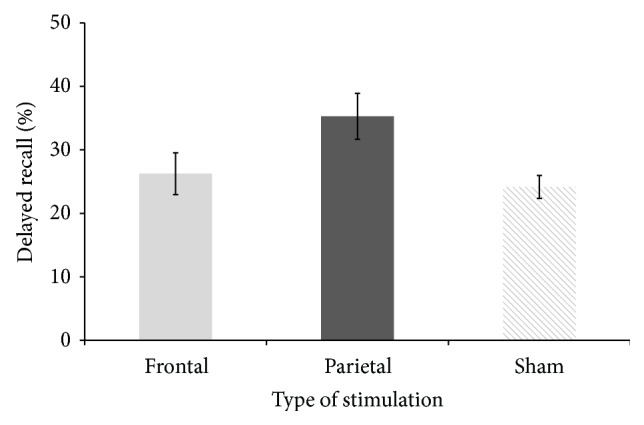
Average percentage of correctly recalled words after seven days (in the second session) across the three types of brain stimulation. Error bars represent the ±SEM.

We also ran a one-way AVOVA on the percentage of correctly recalled words in the second experimental session (seven days later) with stimulation (frontal, parietal, or sham) as an independent variable. We found a significant effect of stimulation (*F*(2, 51) = 3.85, *p* = 0.028) and Fisher's post hoc analysis confirmed that parietal stimulation was associated with better memory performance seven days later than both frontal and sham stimulation (*p* = 0.039 and *p* = 0.012, resp.), while there was no significant difference between frontal and sham stimulation (*p* = 0.63) (see Figure 3).

In the first experimental session, participants were not required to recall the entire list of words during every block, but only the words that were not correctly recalled in the preceding blocks (i.e., drop-out method), while in the second session participants were required to recall all the 40 words. Therefore, a direct comparison between the first and the second experimental sessions would not provide a useful comparison (see also [32]).

## 4. Discussion

The idea of stimulating the brain with electrical current is relatively old (for reviews see [34, 35]), but it has recently been rediscovered in neuroscience and neurorehabilitation [36–40]. Additionally, the recent development of tRNS provided a less detectable and thus more user-friendly tool for brain stimulation [12, 13].

Our results suggest that posterior parietal stimulation produced a long-term advantage in acquisition of foreign vocabulary, thus indicating that the posterior parietal cortex may be implicated in the linguistic network, which has so far been shown to involve areas such as Broca's and Wernicke's areas, the superior and posterior temporal areas, and the auditory cortex [19–24]. Traditionally, the posterior parietal cortex has been considered to be involved in the deployment of spatial attention [41]. More recent studies have shown that it also plays a role in numerical processing [42], in working memory tasks [43], in self-body representation [44], and in self- versus others' representation [45]. Our results suggest that the posterior parietal cortex may also be involved in language processing, which substantiates the findings of previous studies that employed different techniques [24, 26]. Given that, in this study, participants learnt vocabulary of an unknown foreign language, the posterior parietal cortex may specifically be recruited for linguistic tasks involving foreign languages. The extensive anatomical connections between the posterior temporal language areas (e.g., Wernicke's area) and the posterior parietal cortex further support the finding that the latter is recruited for linguistic tasks [28–46]. Here we present the first causal evidence of its role due to the employment of brain stimulation techniques [18].

A final consideration is that our results might clarify the debate on which areas are involved in semantic memory. There is strong evidence that temporal and inferior parietal cortices are involved, whilst evidence on the role of the posterior parietal cortex is sparse [47–49]. In our task participants had to remember semantic links between concepts (garden, food, money, etc.) and words in an unknown language. The results reported here imply that the posterior parietal cortex might participate in the* formation* of these semantic links at the time of learning, thus improving semantic memory performance at the time of the test taken seven days later.

Unexpectedly, we did not find evidence for the involvement of the dorsolateral prefrontal cortex in acquisition of foreign language. Although this area is not traditionally included in the frontotemporal network involved in linguistic processing [19, 20, 22], some studies have suggested otherwise (e.g., [27]). Nevertheless, a careful examination of the literature suggests that DLPFC might be involved in linguistic (and nonlinguistic) tasks requiring behavioural control and inhibition, such as language switch in bilingual individuals [27, 50], which were not employed in our study. Also, its role might be correlational in the processing of linguistic information, but not causally related to the direct acquisition of new vocabulary.

We found that stimulation of the posterior parietal cortex during learning produced a* delayed* benefit in language acquisition that only appeared at the lattertest. Participants in all three groups learnt words at equivalent levels in the first session, whilst participants in the parietal stimulation group performed substantially better than those in the other two groups in the second session. It is likely that differences emerged during the seven days between the two sessions. We infer from these findings that tRNS enhances the memory consolidation processes that occur during sleeping [51–54]. It has been shown that, during sleep, memory may be consolidated by spontaneous reactivation of the neural networks that were active during wakefulness [55–57]. Transcranial random noise stimulation increases neural spontaneous activity in the long term [14, 58, 59]; therefore, when it is applied to relevant cortical areas, it might* facilitate* neural reactivation that occurs during sleep. Nevertheless, long-term effects of microelectric brain stimulation are well known and our results confirm these past findings in the literature [14, 60].

In summary, by using tRNS, we possibly facilitated the “normal” memory consolidation processes that occur* during sleep*, which produced long-term benefit in foreign vocabulary acquisition. Indeed, further research is necessary to explore the interesting notion that tRNS may enhance foreign language acquisition by facilitating memory consolidation during sleep in greater detail, for example, by combining tRNS and sleep deprivation. Moreover, future studies should investigate the role of tRNS in other linguistic features to determine the ecological validity of the present findings. In fact, here we were focusing on the vocabulary or lexicon only, whilst languages possess also syntax and phonetics. Determining whether tRNS could improve the acquisition of every feature of a foreign language would extend the ecological and practical validity of the role of the posterior parietal cortex beyond just vocabulary acquisition demonstrated here.

Nevertheless, for the first time, this study demonstrated that tRNS can be successfully employed to improve long-term acquisition of foreign vocabulary and provides causal evidence for extending the cortical network involved in language processing to posterior parietal cortex. Aside from theoretical importance, these findings are of potential relevance for the wider society. In fact, as discussed in the Introduction, language acquisition is costly (in many terms), but increasingly necessary. Thus, providing a simple and cost-effective method for speeding language acquisition (neuroenhancement) would be particularly advantageous for those individuals with scarce economic resources and/or scarcity of time. This will be likely to increase their chances of finding better jobs [61, 62] and improve society as a whole.

## Figures and Tables

**Table 1:** The Swahili-English word pairs used in the experiment.

Number	Swahili	English
1	Adui	Enemy
2	Bustani	Garden
3	Buu	Maggot
4	Chakula	Food
5	Dafina	Treasure
6	Elimu	Science
7	Embe	Mango
8	Fagio	Broom
9	Farasi	Horse
10	Fedha	Money
11	Goti	Knee
12	Hariri	Silk
13	Kaa	Crab
14	Kaburi	Grave
15	Kaputula	Shorts
16	Leso	Scarf
17	Maiti	Corpse
18	Malkia	Queen
19	Mashua	Boat
20	Ndoo	Bucket
21	Nyanya	Tomato
22	Nyuni	Bird
23	Paka	Cat
24	Pazia	Curtain
25	Pipa	Barrel
26	Pombe	Beer
27	Punda	Donkey
28	Rembo	Ornament
29	Roho	Soul
30	Sala	Prayer
31	Sumu	Poison
32	Tabibu	Doctor
33	Theluji	Snow
34	Tumbili	Monkey
35	Usingizi	Sleep
36	Vuke	Steam
37	Yai	Egg
38	Zeituni	Olives
39	Ziwa	Lake
40	Zulia	Carpet
